# Probiotication of Plum Pulp and Conditions Effects Freeze-Drying in Cell Viability, Functional Properties and Antioxidant Activity

**DOI:** 10.3390/foods13223551

**Published:** 2024-11-07

**Authors:** Mailson Gregório, Morgana Araújo, Aline Albuquerque, Thais Rodrigues, Newton C. Santos, Maria Tereza Fonseca, Maria Eduarda da Costa, Anna Tomé, Josivanda Gomes, Deyzi Gouveia, Hugo M. Lisboa, Ana Paula Rocha

**Affiliations:** 1 Unidade Académica Engenharia Agrícola, Universidade Federal Campina Grande, Av. Aprigio Veloso 882, Campina Grande 58400-900, Brazil; 2 Unidade Académica Engenharia de Alimentos, Universidade Federal Campina Grande, Av. Aprigio Veloso 882, Campina Grande 58400-900, Brazilana_trindade@yahoo.com.br (A.P.R.)

**Keywords:** *Prunus salicina lindl*, *Bifidobacterium animalis* ssp. *lactis*, non-dairy probiotics, antioxidant potential

## Abstract

This study investigated the effects of fermenting plum pulp with *Bifidobacterium animalis* ssp. *lactis* (BAL) on its physicochemical and bioactive properties, as well as the optimization of the freeze-drying (FD) process to develop a fruit-based probiotic delivery system. Fermentation significantly reduced the pH and total acidity of the pulp, achieving a cell viability of 11 log CFU/mL. The FD process was optimized using a factorial design, with maltodextrin concentration (3, 5, and 7%) and freezing temperature (−150, −100, and −50 °C) as variables. The P2 experiment, which used 7% maltodextrin and freezing at −150 °C, showed the best results in terms of yield (25.67%), cell viability (8 log CFU/g), and probiotic survival rate (97.66%). Samples P5, P6, and P7, prepared with 5% maltodextrin and freezing at −100 °C, exhibited the highest levels of bioactive compounds and antioxidant activity (*p* < 0.05). During 28 days of storage, all samples maintained cell viability without significant logarithmic reduction. In summary, probiotic plum powders offer an excellent plant-based alternative for probiotic consumption, providing safe levels of beneficial bacteria and bioactive compounds with antioxidant action, meeting health and nutrition needs.

## 1. Introduction

The use of probiotic microorganisms in plant-based matrices has increased significantly, mainly due to the positive effects of adding functional foods to the human diet. The global production of dairy alternative products, especially plant-based ones, shows continuous growth and is estimated to reach approximately 44.8 million dollars by 2027 [1]. Plant-based matrices play an important role in the development of functional foods, as they contain antioxidant compounds, vitamins, and dietary fibers, which in turn contribute to the delivery of probiotic cultures [2]. The consumption of non-dairy probiotics offers a solution to issues such as high cholesterol levels, allergies, and intolerances associated with dairy product consumption. Additionally, it is a suitable alternative to meet the growing preference for vegan diets [3].

Probiotic bacteria are live microorganisms that, when consumed in adequate amounts in the human diet, can promote health improvements [4]. BAL belongs to the Bifidobacterium genus, which consists of beneficial bacteria used for preventing diarrhea, reducing the risk of constipation, and regulating immune responses [5]. Bifidobacterium and Lactobacillus are the main lactic acid bacteria used in food production; however, BAL has a higher survival rate through various technological processes and also promotes the production of bacteriocins, which are antimicrobial peptides that can be used as food preservatives [6,7].

Although its primary industrial application is related to dairy production, studies have shown that this culture is suitable for and can thrive in fruit pulp matrices, such as umbu-cajá (*Spondias mombin*) [8], blueberry (*Vaccinium* spp.), and blackberry (*Rubus fruticosus*) [9]. This demonstrates that the lactic acid bacteria in question can also be developed in plum pulp (*Prunus subg*. *Prunus*). Additionally, plum pulp contains important nutrients, serving as a source of vitamins and phytochemicals such as phenolics, flavonoids, and anthocyanins, which are associated with antioxidant activity.

Intrinsic factors of the fruit, such as pH and acidity, can reduce the metabolic activity of bacteria during inoculation and fermentation, especially in the case of Bifidobacterium species, which are sensitive to acidic environments, as is common with most fruits. Therefore, it is necessary to adjust the environment to allow proper development of these bacteria. Additionally, factors related to the gastrointestinal tract can also inactivate the bacteria during the natural digestive process. As a result, probiotics require protection against degradation and rapid inactivation until they are delivered to the desired location [10].

Drying is one of the most traditional food preservation methods and significantly reduces the water activity in food, allowing it to be stored for long periods without spoilage. As a gentle dehydration method, FD removes water primarily through sublimation. Food products subjected to FD retain a high content of vitamins and active compounds, ensuring excellent product quality [11,12]. However, the steps preceding FD, such as the selection and concentration of the encapsulating material, as well as the freezing temperature, can also affect the final product quality [13]. Maltodextrin, widely used as an encapsulating agent, plays a key role in preserving bioactive molecules and maintaining cell viability [14]. Therefore, optimization studies are necessary to ensure that the desired properties are maintained during and after the FD process.

In this study, we aimed to develop and optimize a probiotic plum pulp powder by fermenting plum pulp with *Bifidobacterium animalis* subsp. *lactis* (BAL) and applying freeze-drying (FD) techniques. Specifically, we analyzed the effects of varying maltodextrin concentrations and freezing temperatures on yield, probiotic cell viability, BAL survival rate, and the techno-functional properties of the resulting plum pulp powder to establish an effective plant-based probiotic delivery system.

## 2. Materials and Methods

### 2.1. Plum Pulp Processing

Ripe plums (*Prunus subg*. *Prunus*) were purchased from the local market in the city of Campina Grande, Paraíba, Brazil, followed by cleaning and sanitization in a 100 ppm sodium hypochlorite solution for 10 min. To obtain the pulp, it was processed using an industrial pulper (Bonina 670, Itabuna, Brazil). The extracted pulp was stored under refrigeration at a temperature of 2 °C until it was used in the subsequent steps.

### 2.2. Inoculation of BAL in Plum Pulp

Initially, the pH of the plum pulp was adjusted to 7.0 using a sodium hydroxide (NaOH) solution, in accordance with INS 524 regulations [15]. The pulp was then subjected to pasteurization for 20 min at 70 °C in a heated water bath, followed by cooling in an ice bath until it reached 10 °C, with the aim of inactivating enzymes and potentially spoilage and pathogenic microorganisms. The freeze-dried BAL probiotic culture (DELVO^®^PRO LAFTI B94; DSM Food Specialties) was directly inoculated into the pulp, aiming for a final concentration of 0.1% (*w*/*v*). The choice of a 0.1% (*w*/*v*) concentration for *Bifidobacterium animalis* ssp. *lactis* inoculation was made to ensure sufficient probiotic cell density for effective fermentation while avoiding potential inhibition from excessive microbial load in the plum pulp matrix. The entire process of inoculating and handling the probiotic culture was carried out in a sterile, controlled environment to avoid possible contamination of the samples. After inoculation, the samples were incubated at 37 °C for 22 h in a bacteriological incubator (model Solidsteel, São Paulo, Brazil).

### 2.3. Characterization of Fermented Plum Pulp

#### 2.3.1. pH and Total Titratable Acidity

The pH was determined using a pH meter (model DL-PH). Total titratable acidity was assessed using a titrimetric method with 0.1 M NaOH solution and 1% phenolphthalein indicator until the appearance of a pink color. The results were expressed as g of citric acid/100 g, following the AOAC methods [16].

#### 2.3.2. Water Content and Water Activity

Water content was determined gravimetrically in an oven at 105 °C (model luca-80/42, São Paulo, Brazil) until a constant mass was achieved, using an analytical balance (AT-R, Shimadzu, São Paulo, Brazil) and the results were expressed as a percentage (%), in accordance with AOAC [17]. Water activity (aw) was measured using a water activity meter (Decagon 4TE, Aqualab, Pullman, WA, USA) at 25 °C.

#### 2.3.3. Total Phenolic Compounds (TPC)

The TPC was determined using the Folin-Ciocalteu spectrophotometric method, according to Waterhouse [18]. Extracts were obtained by macerating 1.0 g of the sample with 50 mL of distilled water, followed by resting for 30 min and filtration through filter paper. Then, an aliquot of the extract, distilled water, and Folin-Ciocalteu were vortexed and allowed to rest for 5 min. To this mixture, 250 µL of sodium carbonate solution (20%) were added, followed by vortexing and heating in a water bath at 40 °C for 30 min in the absence of light during the assay. The absorbance of the sample was read at a wavelength of 765 nm in a spectrophotometer UV-Vis (Agilent Technologies, Santa Clara, CA, USA; Cary 60). The standard curve was prepared using gallic acid as a standard, at a concentration of 100 μg/mL, and the results were expressed in mg GAE (gallic acid equivalent)/100 g.

#### 2.3.4. Total Tannins (TT)

The TT were determined according to the methodology described by Goldstein and Swain [19], using the tannic acid curve as a standard. Extracts were obtained by macerating 0.5 g of the sample with 25 mL of distilled water, followed by resting for 30 min and filtration through filter paper. Then, an aliquot of the extract, distilled water, and Folin-Ciocalteu were vortexed and allowed to rest for 5 min. To this mixture, 250 µL of sodium carbonate solution (20%) were added, followed by vortexing and heating in a water bath at 40 °C for 30 min in the absence of light during the assay. The absorbance of the sample was read at a wavelength of 725 nm in a spectrophotometer UV-Vis (Agilent Technologies; Cary 60), and the results were expressed in mg TAE (tannic acid equivalent)/100 g.

#### 2.3.5. Total Carotenoids (TC)

The total carotenoid content was quantified according to the method of Lichtenthaler [20]. In summary, 200 mg of each sample were gently ground with 0.2 g of calcium carbonate, dispersed in 10 mL of 80% acetone, and centrifuged for 10 min at 3000 rpm and 10 °C. The supernatants were collected, and absorbance were measured at 470 nm using a spectrophotometer UV-Vis (Agilent Technologies; Cary 60). The results were expressed in milligrams of carotenoids per 100 g of sample (mg/100 g).

#### 2.3.6. Total Flavonoids (TF) and Anthocyanins (TA)

The total content of flavonoids and anthocyanins was determined using the method of Francis & Markakis [21]. Initially, extracts containing 500 mg of the samples were prepared, which were manually macerated with a solution of 95% ethanol + 1.5 N HCl (85:15 *v*/*v*). The resulting extracts were transferred to Falcon tubes and stored at 10 °C for 24 h. After this period, the tubes were centrifuged for 10 min at 3000 rpm and 10 °C. The supernatants were then collected, and absorbances were measured at 374 nm for flavonoids and 535 nm for anthocyanins using a spectrophotometer UV-Vis (Agilent Technologies; Cary 60). The results were expressed in milligrams per 100 g of sample (mg/100 g).

#### 2.3.7. Cell Viability

Cell viability was determined by counting bacteria in serial dilution in 0.1% (*w*/*w*) sterile peptone water, where aliquots were inoculated on MRS agar and incubated at 37 °C for 72 h in anaerobic jars with an oxygen removal system (Interlab, São Paulo, Brazil), followed by counting colony-forming units (CFU/g) according to the International Dairy Federation [22]. Confirmatory tests were also performed to verify the identity and viability of *Bifidobacterium animalis* ssp. *lactis* in alignment with this standard.

### 2.4. FD Process of Probiotic Plum Pulp

To dry the probiotic plum pulp by FD, maltodextrin DE 10 (Mor-Rex^®^ 1910, São Paulo, Brazil) was initially added as a drying aid in different concentrations (3%, 5%, and 7%) according to the factorial design matrix. This was followed by freezing in cryogenic freezers (Model IULT 9504D, INDREL^®^, Paraná, Brazil) at various freezing temperatures (−150, −100, and −50 °C) for 24 h (conditions defined based on preliminary tests. The frozen samples were then freeze-dried in a benchtop lyophilizer (model LIOTOP-L101, São Carlos, Brazil) at −48 °C and a pressure of 11.58 mbar for 72 h.

The experimental conditions were carried out according to the 22 factorial design matrix, consisting of 4 factorial points (levels ± 1) and 3 central points (level 0), totaling 7 points (Table 1). The freeze-dried material was ground using an electric processor (Philips, CHOP RI7300, São Paulo, Brazil) and packaged in flexible laminated containers. The dependent variables of the factorial design matrix were analyzed concerning yield, cellular viability (Section 2.3.7), and survival rate according to the following methodologies.

#### 2.4.1. Process Yield

The yield was calculated by the ratio of the mass of solids in the powders collected at the end of drying to the initial mass of solids present in the sample before drying (Equation (1)).

(1) $$Yield\left(\%\right)=\frac{Mass\;of\;collected\;solids\;(g)}{Initial\;mass\;of\;solids\;(g)}\times 100$$

#### 2.4.2. Survival Rate

The survival rate of BAL was calculated to predict the efficiency of microencapsulation by FD (Equation (2)) [23].

(2) $$Survival\;rate\left(\%\right)=\frac{\mathrm{Log}N}{\mathrm{Log}N_{0}}\times 100$$

where: Log *N* is the number of viable cells in the powder after FD (CFU/g dry basis), and Log *N*_0_ is the number of viable cells before the FD process (CFU/g dry basis).

### 2.5. In Vitro Antioxidant Activity

#### 2.5.1. Free Radical Scavenging by DPPH (1,1-Diphenyl-2-picrylhydrazyl)

The DPPH assay was conducted as described by Rufino et al. [24]. Initially, 2.4 mg of DPPH were dissolved in 100 mL of pure methanol to prepare a stock solution. Then, a DPPH solution at 6 × 10^−5^ mol/L was prepared by diluting 6 mL of the DPPH stock solution in 100 mL of methanol, which served as a control. Subsequently, 0.1 mL of the sample was mixed with 3.9 mL of the DPPH solution at 6 × 10^−5^ mol/L and thoroughly shaken. This mixture was then incubated in the dark at room temperature for at least 30 min. The colorimetric determination was performed at a wavelength of 515 nm using a spectrophotometer (Agilent Technologies; Cary 60), and the DPPH radical scavenging activity was calculated according to the methodology described by Rufino et al. [24].

#### 2.5.2. 2,2′-Azino-bis(3-ethylbenzothiazoline-6-sulfonic acid (ABTS)

The ABTS assay was conducted according to the methodology proposed by Rufino et al. [24]. Initially, 5 mL of ABTS stock solution and 88 μL of potassium persulfate in deionized water were separately prepared. This mixture was incubated in the dark for at least 16 h to generate the ABTS+ radical. Then, the radical was diluted in ethyl alcohol to reach an absorbance of 0.70 nm ± 0.05 nm at 734 nm. In a dark environment, 30 μL of the extract dilution was mixed with 3.0 mL of the ABTS·+ radical and shaken on a tube shaker, followed by incubation at room temperature for 6 min. The absorbance was then measured at 734 nm using a spectrophotometer (Agilent Technologies; Cary 60), with ethyl alcohol used as a blank. The percentage inhibition of the ABTS radical was calculated according to Rufino et al. [24].

#### 2.5.3. Antioxidant Activity by Iron Reducing Power (FRAP)

In the absence of light, the FRAP reagent was prepared by combining 25 mL of an acetate buffer solution (300 mM/L; pH 3.6), 2.5 mL of FeCl3 (20 mM), and 2.5 mL of the TPTZ solution (10 mM/L of 2,4,6-tris (2-pyridyl)-s-triazine in 40 mM HCl). Then, a 90 μL sample of each flour extract was diluted in 2.7 mL of the FRAP reagent. These mixtures were then combined in tubes and incubated for 30 min in a thermodigestor block (Bioplus IT-2002, Barueri, São Paulo, Brazil). After incubation, absorbance was measured at 595 nm. All absorbance readings were performed using a spectrophotometer (Agilent Technologies; Cary 60) [25].

### 2.6. Viability During Storage

The samples were packaged in laminated containers and stored in a BOD incubator at a temperature of 25 °C for 28 days, protected from any exposure to light. The viability of *Bifidobacterium animalis* spp. was analyzed at 7-day intervals.

### 2.7. Statistical Analysis

The mean data for the physicochemical composition and physical properties were subjected to analysis of variance (ANOVA) using the F test, coefficient of determination and adjusted coefficient of determination (R^2^ and adj R^2^) and significant differences between the means were compared using Tukey’s test at a 5% significance level, with the assistance of Statistic software v. 7.0 (Tibco, Statistica, Palo Alto, CA, USA).

## 3. Results

### 3.1. Physicochemical, Bioactive, and Cellular Viability Characterization of Probiotic Plum Pulp

The composition of fresh plum pulp and fermented plum pulp with probiotics was analyzed to evaluate its potential as a probiotic product, as well as any possible adverse effects from the addition of BAL. The values obtained from the physicochemical characterization and the analysis of bioactive compounds in the plum pulp, both before and after the fermentation process, along with the cellular viability of the probiotics, are presented in Table 2.

The plum pulp, like most fruits, has an acidic pH, which can influence the fermentation process since probiotic bacteria are sensitive to acidic environments. Therefore, neutralization of the pulp is necessary before fermentation. In this study, the fresh pulp showed a pH of 3.36, a value that significantly differed from that of the fermented pulp (*p* < 0.05). Silva & Kunigk [26] also found similar results when analyzing the physicochemical characteristics of whole plum pulp. The total acidity showed an inverse behavior to that of pH, with the fresh pulp presenting a higher concentration of organic acids (1.32 g of citric acid/100 g) compared to the fermented samples (*p* < 0.05). This behavior can be attributed to the heterofermentative metabolism of *Bifidobacterium* spp. bacteria, which produce both acetic acid and lactic acid during fermentation, in contrast to lactobacilli, which produce only lactic acid [27].

The water content and water activity of the plum pulp, before and after the fermentation process, showed high levels, with values exceeding 80% and 0.9, respectively, without statistically significant differences (*p* > 0.05). According to Santos et al. [28], these elevated levels of water content and water activity compromise the stability of the fruits, especially during storage, as they favor the growth of pathogenic and deteriorating microorganisms and accelerate various undesirable chemical reactions. These factors highlight the need to apply efficient preservation techniques, such as lyophilization, which significantly reduces water content, prolonging the shelf life of the product while preserving its nutritional and bioactive properties.

The fermentation of plum pulp with BAL resulted in a significant increase in the levels of the analyzed bioactive compounds, including total phenolic compounds (TPC), total tannins (TT), total flavonoids (TF), total anthocyanins (TA), and total carotenoids (TC) (Table 2) (*p* < 0.05). This increase can be attributed to the action of microbial enzymes released during the fermentation process, which could degrade complex phenolic compounds into simpler, more bioavailable forms, contributing to the higher concentration of these compounds after fermentation [29]. Although both tannins and phenolic compounds play a positive role in the fermentation of fruits, acting as potential antioxidant agents, some studies suggest that at elevated concentrations, these compounds may hinder cell viability in the early stages of fermentation. This occurs because their strong antioxidant action can damage probiotic cells [30], which could be a limitation of the process. However, the results observed in this study indicate that, under the conditions employed, the balance between antioxidant activity and microbial growth was favorable, confirming that fermentation with BAL is an effective approach to enhance the levels of bioactive compounds in plum pulp. This process not only enhances the functional properties of the pulp but may also contribute to the production of functional foods with greater nutritional value.

According to the criteria established by the FAO/WHO, probiotic products must contain a minimum of 10^6^ to 10^7^ CFU/mL of microorganisms during their shelf life to be considered truly probiotic. The fermented plum pulp showed a concentration of viable cells of BAL of 2.98 × 10^11^ CFU/mL. Soares et al. [31], studying the viability of BAL in different matrices, obtained a lower value in whole orange juice with an initial concentration of 7.3 log CFU. Monteiro et al. [32] achieved a maximum concentration of 5.59 × 10^11^ CFU/mL of viable cells of *Lactobacillus reuteri* in passion fruit juice under different fermentation temperature and pH conditions. Lower values were obtained by Nguyen et al. [33] in probiotic pineapple juice fermented with *Bifidobacterium*. A similar result was obtained by Bujna et al. [34] in the development of a probiotic beverage from fermented apricot juice with *Bifidobacterium*. The growth of BAL in plum pulp demonstrates the bacteria’s ability to adapt to non-dairy substrates, as well as offering promising opportunities for the development of innovative fruit-based probiotic products.

### 3.2. Optimization of the FD Process of Probiotic Plum Pulp

FD as a preservation technique provides an alternative and advanced method for utilizing fruit pulps in the production of new probiotic foods. This technique can be employed to convert samples into stable powders with an extended shelf life [35]. Therefore, in this study, different operational conditions of the FD process for probiotic plum pulp were investigated to assess their effects on process yield, cell viability, and survival rate of the probiotic bacteria. The results obtained from the seven experiments are presented in Table 1.

The analysis of the drying process and the quality of the dehydrated product considers not only the viability of the probiotic culture but also the yield as a fundamental parameter for process efficiency. In this study, the yield was directly influenced by the concentration of the drying aid used and the freezing temperature. The highest yield was observed in experiment P2 (25.67%), which used 7% maltodextrin and a freezing temperature of −150 °C. The higher concentration of maltodextrin likely promoted the formation of a denser protective matrix around the probiotic cells during drying, minimizing losses and increasing the final yield. Additionally, the extremely low freezing temperature (−150 °C) contributed to the formation of smaller and more uniform ice crystals, which better preserved cell integrity and matrix structure, facilitating the drying process. These combined conditions resulted in greater retention of the material, leading to higher yield [36]. Previous studies confirm that both the concentration of wall agents, such as maltodextrin, and the freezing temperature play a crucial role in FD efficiency, improving cell protection and process yield [37].

The cell viability results, presented in Table 1, show a variation between 2.32 and 2.58 log CFU/g across experiments P2 and P3. In all experiments, the freeze-dried powders achieved counts exceeding 6 log CFU/g, qualifying them as probiotic foods according to FAO/WHO standards (minimum of 10^6^ to 10^7^ CFU/g). The combination of higher maltodextrin concentrations with ultra-low freezing temperatures resulted in higher viable cell counts. This increase can be explained by the protective role of maltodextrin during the FD process, as it acts as a cryoprotectant, forming a matrix around the probiotic cells that minimizes damage caused by freezing and subsequent water removal [38]. When used in adequate concentrations, maltodextrin helps reduce osmotic stress and prevents the formation of large ice crystals, which can rupture cell membranes, thus preserving probiotic cell viability [39].

Additionally, the use of extremely low freezing temperatures, as in experiment P2 (−150 °C), contributed to the formation of smaller and more uniform ice crystals, which helped preserve cellular structure and facilitated water removal during FD. Lower freezing temperatures result in a more controlled crystallization process, reducing physical damage to probiotic cells and increasing their viability [40]. Studies by Wang et al. [40] corroborate that both the concentration of maltodextrin and freezing temperature are critical variables for maintaining the integrity and functionality of probiotic cells during FD, improving cell viability in the final product.

The survival rate of probiotic bacteria after FD exceeded 70% in all experiments, with the highest survival rates observed in experiments using higher concentrations of maltodextrin (i.e., 7%), regardless of freezing temperature. Survival rates reached 97.66% (P2) and 97.49% (P4), confirming the crucial role of maltodextrin in protecting probiotic cells during FD. Maltodextrin acts as an effective cryoprotectant, forming a protective matrix that minimizes physical damage to cells and preserves their integrity. By limiting the formation of large ice crystals, which could rupture cell membranes, maltodextrin ensures higher cell viability after the drying process [38].

Previous studies, such as those by Viveker et al. [41], which evaluated Sohiong probiotic microcapsules obtained by spray drying, reported lower survival rates compared to FD, highlighting the efficiency of this method in preserving probiotic cultures. Additionally, Nami et al. [42] observed efficiency rates above 98.4% when studying the microencapsulation of *Lactococcus lactis* ABRIINW-N19 in freeze-dried orange juice, corroborating the results of this study, where the high concentration of maltodextrin provided excellent protection to the probiotic cells. Although extremely low freezing temperatures, such as those used in experiment P2 (−150 °C), contributed to minimizing physical damage to the cells due to the formation of smaller ice crystals, the results show that the concentration of maltodextrin was the main protective factor, with high survival rates even at more moderate freezing temperatures. This highlights the importance of adjusting the concentration of cryoprotective agents, such as maltodextrin, to maximize the survival rate during the FD of probiotic products.

The analysis of the determination coefficients (R^2^) and adjusted coefficients (adj R^2^) demonstrated that the regression model used showed a satisfactory fit, with R2 values exceeding 97% and adj R^2^ values above 93%, as presented in Table 1. These values indicate a strong correlation between the experimental data and the variables analyzed, suggesting that the chosen model is suitable for describing the observed responses in the experiment. According to Azmi et al. [43], high R^2^ and adj R^2^ values indicate good accuracy in modeling, which supports the robustness of the factorial design used.

**Figure 1:**
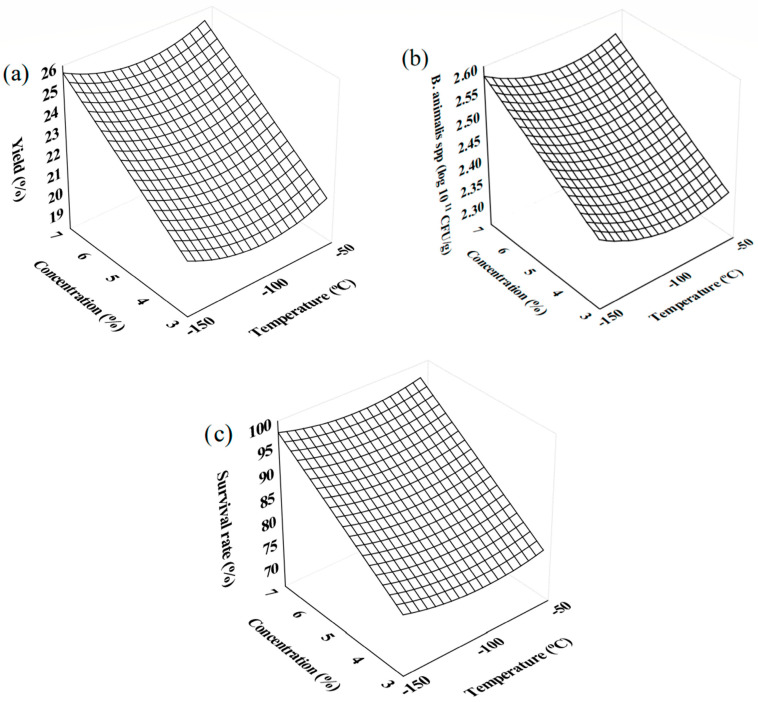
Response surface for: (**a**) yield, (**b**) cell viability, and (**c**) survival rate of probiotics from powdered probiotic plum pulp.

The *p*-values, all below 0.05 for the response variables, indicate that the factors studied had statistically significant effects on the dependent variables, reinforcing the validity of the conclusions drawn from the model. The relationship between the calculated F-value and the tabulated F-value was also analyzed, with the highest ratio observed for the survival rate variable (28.50), showing that this variable was strongly influenced by the experimental conditions. From the response surfaces (Figure 1), it is evident that the best results for yield, cell viability, and survival rate were achieved in experiment P2, which used 7% maltodextrin and freezing at −150 °C. These results can be explained by the higher concentration of maltodextrin, which provided more effective protection to the BAL during the FD process, preventing structural damage and preserving cell viability. The extremely low freezing temperature also favored the formation of smaller ice crystals, which minimized the physical impact on the cells during freezing and subsequent drying. The combination of these factors can be considered efficient and rational strategies for preserving the viability of probiotic cultures during the FD of plum pulp.

### 3.3. Water Content and Water Activity of Probiotic Plum Pulp Powder

The results for water content and water activity (aw) (Table 3) clearly demonstrate that, regardless of the maltodextrin concentration or freezing temperature, all freeze-dried probiotic plum pulp powders exhibited significant stability. The water content of the samples consistently remained below 3.76% (*p* > 0.05), reflecting the effectiveness of the FD process in removing free water, which is a crucial factor for product longevity during storage. Moreover, the aw of the samples ranged from 0.220 (P1) to 0.228 (P3) (*p* > 0.05), staying within a range that prevents microbial spoilage and enzymatic activity responsible for lipid oxidation, as suggested by Janiszewska-Turak et al. [44]. In comparison, studies such as Rishabh et al. [45] reported significantly higher aw values, such as 0.479 in probiotic powders derived from carrot juice, highlighting the effectiveness of the method employed in this study to achieve a more stable product. Therefore, these results confirm that the freeze-dried samples have strong potential for extended storage.

### 3.4. Bioactive Compounds of Probiotic Plum Pulp Powder

The total phenolic content (TPC), total tannins (TT), flavonoids, anthocyanins, and carotenoids of the probiotic plum pulp powder obtained under different FD conditions are shown in Table 3. The results for TPC and TT revealed significant variations (*p* < 0.05) that can be attributed to both the maltodextrin concentration and the freezing temperature. Samples P5, P6, and P7, prepared with 5% maltodextrin and frozen at −100 °C, showed the highest concentrations of these compounds. Notably, sample P5 recorded the highest levels of TPC (9615.77 mg GAE/100 g) and TT (6764.24 mg/100 g), with statistically significant differences (*p* < 0.05). According to Aryaee et al. [46], the high retention of phenolic content can also be attributed to the FD process, where the absence of heat and the formation of ice crystals break down cell structures, facilitating the extraction of phenolic compounds.

Similarly, the samples showed significant increases (*p* < 0.05) in flavonoid, anthocyanin, and carotenoid levels compared to the fermented pulp before drying. Flavonoid levels ranged from 83.87 mg/100 g (P2) to 120.98 mg/100 g (P7), reflecting the concentration effect of water removal during FD. Anthocyanins, antioxidant compounds responsible for the pulp’s color, also increased, rising from 9.26 mg/100 g in the fermented pulp to values between 16.26 mg/100 g (P2) and 35.54 mg/100 g (P7). Carotenoids, on the other hand, showed wider variations, ranging from 42.20 µg/100 g (P2) to 96.71 µg/100 g (P5) (*p* < 0.05) after FD. This behavior suggests that while some carotenoids may be sensitive to oxidation during the drying process, the combination of freezing at −100 °C and the optimal maltodextrin concentration in P5 was effective in preserving and stabilizing these compounds.

Our results highlight the crucial role that the 5% maltodextrin concentration played in protecting phenolic compounds during the FD process. Maltodextrin acts as a cryoprotectant by forming a matrix around bioactive compounds, reducing degradation from factors such as heat or oxidation during drying. This matrix helps stabilize phenolic compounds like TPC, TT, flavonoids, anthocyanins, and carotenoids, preserving their concentrations. When the maltodextrin concentration is lower (3%, as in samples P1 and P3), the protective matrix is not sufficiently robust, leading to greater losses of bioactive compounds during FD. Conversely, a higher maltodextrin concentration (7%, as in P2 and P4) may increase the solution’s viscosity, hindering the formation of an appropriate structure that promotes efficient drying and compound preservation. This explains why samples with 7% maltodextrin had lower concentrations of these compounds. Additionally, the ultra-low freezing temperature minimized degradation and mechanical stress, resulting in samples with significantly higher bioactive compound contents. According to Ma et al. [47], faster freezing rates, i.e., processes subjected to ultra-low temperatures, tend to cause the release of bound bioactive compounds in correlation with the cell wall.

### 3.5. Antioxidant Activity of Probiotic Plum Pulp Powder

**Figure 2:**
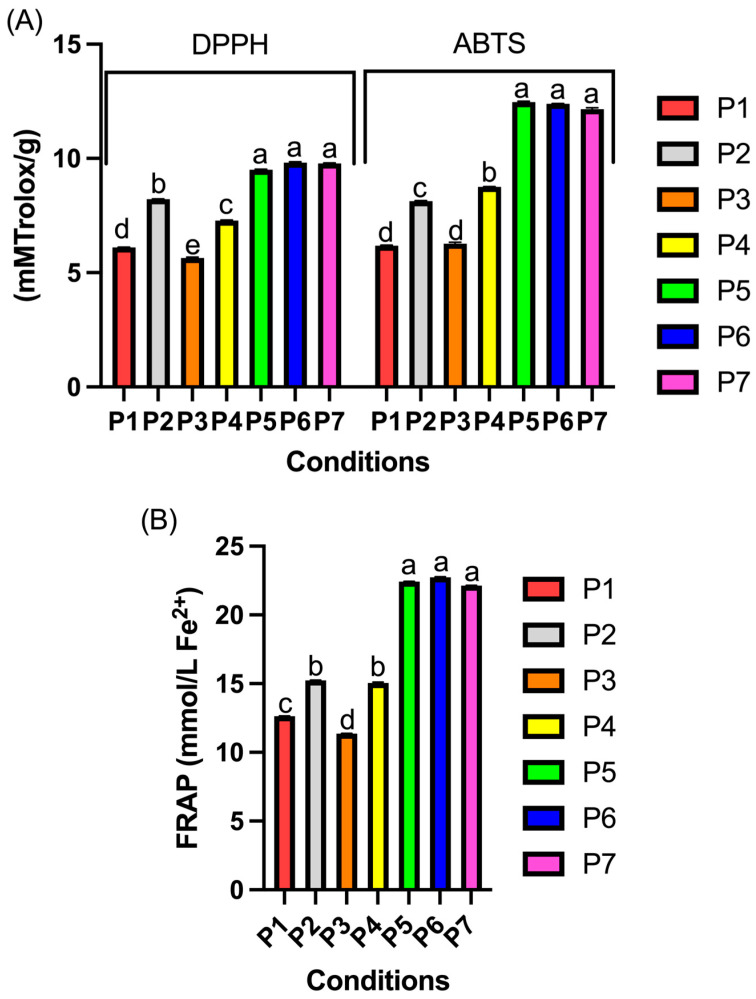
In vitro antioxidant activity of powdered probiotic plum pulp by DPPH and ABTS methods (**A**) and FRAP (**B**). Lowercase letters (a, b, c, d) indicate statistical differences (*p* < 0.05) between different conditions.

The antioxidant activity of probiotic plum powder was determined using the DPPH, ABTS, and FRAP assays, and the results are presented in Figure 2.

The antioxidant activity in the powders obtained under different conditions revealed significant variations in values (*p* < 0.05). Samples P5, P6, and P7, with 5% maltodextrin and frozen at −100 °C, showed the highest antioxidant activity values, with ABTS ranging from 12.11 to 12.44 mM Trolox/g, DPPH from 9.52 to 9.85 mM Trolox/g (Figure 2A), and FRAP from 22.15 to 22.76 mmol Fe^2+^/g (Figure 2B). This high antioxidant capacity can be attributed to the higher content of phenolic compounds, such as flavonoids and anthocyanins, which have strong antioxidant activity. The concentration of 5% maltodextrin provided efficient protection to the bioactives during lyophilization, preserving their activity, while the low freezing temperature (−100 °C) helped to prevent the thermal and oxidative degradation of these compounds due to the formation of smaller ice crystals, which minimized cellular stress.

On the other hand, samples P1 and P3, with only 3% maltodextrin, exhibited lower antioxidant capacity. The lower concentration of maltodextrin did not provide adequate protection against the degradation of bioactive compounds during lyophilization, resulting in lower retention of flavonoids and anthocyanins, which are the main contributors to antioxidant activity. Additionally, the higher freezing temperature (−150 °C) used in P1 and P3 may have contributed to a greater formation of larger ice crystals, causing more structural damage to the cells, thereby promoting the loss of antioxidant compounds. Thus, both the amount of maltodextrin and the freezing temperature significantly influenced the preservation of antioxidant activity in the samples, making them promising for applications in functional food products, where maintaining antioxidant activity is essential for providing health benefits during prolonged storage.

### 3.6. Probiotic Viability During Storage

**Figure 3:**
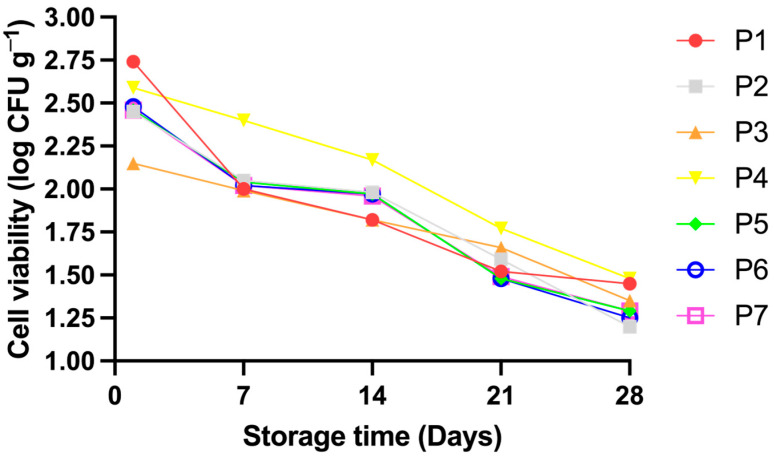
Cell viability of the probiotic over 28 days of storage at 25 °C.

The viability of probiotics is directly related to the characteristics of the food matrix and the added microorganisms, as well as the interactions between them [29]. In this study, the viability of BAL encapsulated in plum pulp through FD was evaluated over 28 days of storage at 25 °C, as shown in Figure 3.

Although there was a significant reduction in the viability of probiotic strains in all formulations (*p* < 0.05), which is expected due to the impact of time and environmental conditions, it is important to highlight that formulation P4 exhibited the lowest reductions in viability throughout storage, suggesting greater stability of the probiotic in this formulation.

The results confirm that the FD process was effective, as all samples maintained viability counts above 11 log CFU/g during the 28 days, with no significant reduction in the logarithmic cycle. This indicates that probiotics encapsulated in plum pulp can continue to offer health benefits, such as promoting gut microbiota balance and improving immune functions, even after prolonged storage [48].

When comparing our results with those of Sultana et al. [49], who investigated the viability of *Lactobacillus casei* in different encapsulation forms in orange juice over 30 days, we observed superior performance in our plum pulp microcapsules. In the study by Sultana et al. [49], there was a more pronounced logarithmic reduction, with free *L. casei* not maintaining the minimum viability (6–7 log CFU/mL) after 24 days of storage, which would compromise the expected probiotic effects. In contrast, the plum pulp microcapsules maintained significantly higher viability over time, demonstrating their ability to protect probiotics and prolong their efficacy during storage. These results reinforce the potential of plum pulp microcapsules as an efficient matrix for probiotic encapsulation, ensuring the necessary viability for microorganisms to exert their beneficial effects even under prolonged storage conditions.

## 4. Conclusions

This study demonstrated that the fermentation of plum pulp with BAL positively influenced the physicochemical and bioactive properties of the pulp. The fermentation resulted in a significant reduction in pH and total acidity, indicating the effective action of probiotic bacteria in metabolizing sugars and producing organic acids. Furthermore, the study presented an optimized FD process, highlighting new perspectives for developing a fruit-based probiotic delivery system. All analyzed variables had a significant impact on the observed responses. Experiment P2, which used 7% maltodextrin and freezing at −150 °C, revealed the best results in terms of yield, cellular viability, and survival rate of probiotic bacteria. On the other hand, samples P5, P6, and P7, prepared with 5% maltodextrin and freezing at −100 °C, stood out for having the highest levels of bioactive compounds and the greatest antioxidant activity. Additionally, during the 28-day storage period, all samples maintained cellular viability, with no significant reduction in the logarithmic cycle. These results indicate that the system developed in this study can be used for the production of healthy fruit-based foods and probiotics, enriched with antioxidant phenolic compounds.

## Figures and Tables

**Table 1:** Operational conditions of the freeze-drying process for probiotic plum pulp and their effects on yield, cell viability, and survival rate. Each experimental condition (P1–P7) was performed with one replicate.

Experiments	T (°C)	Concentration (%)	Yield (%)	Cell Viability (11 log CFU/g)	Survival Rate (%)
P1	−150 (−1)	3 (−1)	19.51	2.36	73.01
P2	−150 (−1)	7 (+1)	25.67	2.58	97.66
P3	−50 (+1)	3 (−1)	19.36	2.32	72.10
P4	−50 (+1)	7 (+1)	25.65	2.56	97.49
P5	−100 (0)	5 (0)	22.18	2.44	85.15
P6	−100 (0)	5 (0)	22.25	2.46	85.32
P7	−100 (0)	5 (0)	22.20	2.41	85.33
ANOVA					
R^2^			99.98	97.28	99.99
Adj R^2^			99.98	93.18	99.99
*p*-value			<0.0008	<0.0117	<0.00114
Fc/Ft			5.56	1.42	28.50

**Table 2:** Water content, water activity, bioactive compounds, and cell viability of probiotic plum pulp.

Parameters	Fresh Pulp	Fermented Pulp
pH	3.36 ± 0.05 ^b^	5.87 ± 0.03 ^a^
Total titratable acidity (g of citric acid/100 g)	1.32 ± 0.09 ^a^	0.73 ± 0.04 ^b^
Water content (%)	87.55 ± 0.40 ^a^	87.18 ± 0.29 ^a^
Water activity, aw	0.98 ± 0.00 ^a^	0.98 ± 0.00 ^a^
TPC (mg GAE/100 g)	1775.33 ± 2.67 ^b^	2259.11 ± 1.80 ^a^
TT (mg TAE/100 g)	724.65 ± 1.73 ^b^	1145.53 ± 2.07 ^a^
TF (mg/100 g)	39.67 ± 0.55 ^b^	44.85 ± 0.26 ^a^
TA (mg/100 g)	3.64 ± 0.15 ^b^	9.26 ± 0.23 ^a^
TC (µg/100 g)	48.94 ± 0.34 ^a^	72.97 ± 0.19 ^a^
Cell viability (CFU/mL)	N/D	2.98 × 10^11^

Legend: TPC: Total Phenolic Compounds (mg GAE/100 g); TT: Total tannins (mg TAE/100 g); TF: Total flavonoids (mg/100 g); TA: Total anthocyanins (mg/100 g); TC: Total carotenoids (µg/100 g); N/D: not determined; Means with different superscript lowercase letters are significantly different as determined by a one-way ANOVA test (*p* < 0.05).

**Table 3:** Water content, water activity, and bioactive compounds of powdered probiotic plum pulp obtained under different freeze-drying conditions. Measurements were taken in triplicate.

Experiments	Water Content (%)	Water Activity	TPC (mg GAE/100 g)	TT (mg TAE/100 g)	TF (mg/100 g)	TA (mg/100 g)	TC (µg/100 g)
P1	3.46 ± 0.03 ^b^	0.220 ± 0.01 ^a^	1002.05 ± 0.03 ^g^	5218.28 ± 0.03 ^e^	92.04 ± 0.03 ^c^	24.61 ± 0.03 ^f^	72.98 ± 0.22 ^c^
P2	3.49 ± 0.05 ^b^	0.224 ± 0.00 ^a^	8958.56 ± 0.01 ^d^	5079.46 ± 0.03 ^f^	83.87 ± 0.01 ^e^	16.26 ± 0.02 ^g^	42.20 ± 0.12 ^e^
P3	3.76 ± 0.01 ^a^	0.228 ± 0.02 ^a^	1095.11 ± 0.01 ^f^	5378.04 ± 0.01 ^d^	95.46 ± 0.02 ^b^	27.12 ± 0.04 ^e^	72.97 ± 0.11 ^c^
P4	3.51 ± 0.04 ^b^	0.224 ± 0.01 ^a^	8355.12 ± 0.04 ^e^	5020.36 ± 0.06 ^g^	91.17 ± 0.01 ^d^	29.44 ± 0.01 ^d^	44.45 ± 0.14 ^d^
P5	3.55 ± 0.03 ^b^	0.227 ± 0.01 ^a^	9615.77 ± 0.05 ^b^	6764.24 ± 0.04 ^a^	120.81 ± 0.20 ^a^	34.70 ± 0.01 ^a^	96.71 ± 0.05 ^a^
P6	3.43 ± 0.02 ^b^	0.221 ± 0.01 ^a^	9588.8 ± 0.02 ^a^	6703.34 ± 0.06 ^c^	120.28 ± 0.35 ^a^	35.47 ± 0.03 ^c^	95.84 ± 0.23 ^b^
P7	3.54 ± 0.01 ^b^	0.225 ± 0.02 ^a^	9506.73 ± 0.03 ^c^	6750.02 ± 0.02 ^b^	120.98 ± 0.05 ^a^	35.54 ± 0.02 ^b^	95.49 ± 0.31 ^b^

Legend: Mean ± standard deviation; TPC: Total Phenolic Compounds (mg GAE/100 g); TT: Total tannins (mg TAE/100 g); TF: Total flavonoids (mg/100 g); TA: Total anthocyanins (mg/100 g); TC: Total carotenoids (µg/100 g); Means followed by lowercase equal letters in the same column do not differ significantly at 0.05 probability level between experiments.

## Data Availability

The original contributions presented in the study are included in the article, further inquiries can be directed to the corresponding author.
